# Molecular Phylogeography and Evolutionary History of *Poropuntius huangchuchieni* (Cyprinidae) in Southwest China

**DOI:** 10.1371/journal.pone.0079975

**Published:** 2013-11-25

**Authors:** Xiaoyun Wu, Jing Luo, Song Huang, Ziming Chen, Heng Xiao, Yaping Zhang

**Affiliations:** 1 Laboratory for Conservation and Utilization of Bio-resource, Key Laboratory for Animal Genetic Diversity and Evolution of High Education in Yunnan Province, School of Life Sciences, Yunnan University, Kunming, Yunnan, China; 2 State Key Laboratory of Genetic Resources and Evolution, Kunming Institute of Zoology, Chinese Academy of Sciences, Kunming, Yunnan, China; 3 Key Laboratory of Animal Models and Human Disease Mechanisms, Chinese Academy of Sciences and Yunnan Province, Kunming Institute of Zoology, Chinese Academy of Sciences, Kunming, Yunnan, China; 4 Kunming Zoo, Kunming, Yunnan, China; University of Connecticut, United States of America

## Abstract

**Background:**

The evolution of the Yunnan Plateau’s drainages network during the Pleistocene was dominated by the intense uplifts of the Qinghai-Tibetan Plateau. In the present study, we investigated the association between the evolutionary histories of three main drainage systems and the geographic patterns of genetic differentiation of *Poropuntius huangchuchieni*.

**Methodology/Principal Findings:**

We sequenced the complete sequences of mitochondrial control region for 304 specimens and the sequences of Cytochrome *b* gene for 15 specimens of the species *P. huangchuchieni* and 5 specimens of *Poropuntius opisthoptera*. Phylogenetic analysis identified five major lineages, of which lineages MK-A and MK-B constrained to the Mekong River System, lineages RL and LX to the Red River System, and lineage SW to the Salween River System. The genetic distance and network analysis detected significant divergences among these lineages. Mismatch distribution analysis implied that the population of *P. huangchuchieni* underwent demographic stability and the lineage MK-B, sublineages MK-A1 and LX-1 underwent a recent population expansion. The divergence of the 5 major lineages was dated back to 0.73–1.57 MYA.

**Conclusions/Significance:**

Our results suggest that *P. opisthoptera* was a paraphyletic group of *P. huangchuchieni*. The phylogenetic pattern of *P. huangchuchieni* was mostly associated with the drainage’s structures and the geomorphological history of the Southwest Yunnan Plateau. Also the differentiation of the major lineages among the three drainages systems coincides with the Kunlun-Yellow River Movement (1.10–0.60 MYA). The genetic differentiation within river basins and recent demographical expansions that occurred in some lineages and sublineages are consistent with the palaeoclimatic oscillations during the Pleistocene. Additionally, our results also suggest that the populations of *P. huangchuchieni* had keep long term large effective population sizes and demographic stability in the recent evolutionary history, which may be responsible for the high genetic diversity and incomplete lineages sorting of *Poropuntius huangchuchieni.*

## Introduction

Past tectonic movements and climatic changes have greatly shaped the structure of hydrographic systems [1], [2], [3]. Likewise, since freshwater fish species are strictly confined to freshwater drainages, the historical connections, capture, reversal and separation of rivers are a driving force behind their diversification and speciation [1], [4], [5], [6], [7]. Consequently, the genetic structure and the dispersal of freshwater fish species is also strongly connected to historical and ecological changes to the aquatic environment [1], [4], [5], [6], [7], so by examining the historical biogeography of freshwater fishes could provide a natural link in understanding concurrent geographical and biotic evolution of a given region [8].

As the southeastern neighbor of Qinghai–Tibetan Plateau, the Yunnan Plateau has consistently responded to each period of the Qinghai–Tibetan Plateau’s uplifting, which reached its present elevation some 3.4 MYA, during the Pliocene. The dramatic uplifting of Yunnan Plateau at the same time not only dramatically re-shaped the composition and configurations of geographic and aquatic environments, but also greatly changed the region’s climate [9], [10]. The three big rivers of the Yunnan Plateau–the Mekong, Salween, and Red Rivers–also experienced remarkable changes to their drainage networks during these geologic movements [11]. These movements had a markedly different influence in shaping the geomorphologic configurations of each different region of the Yunnan Plateau. As such, the configuration of the modern rivers differs significantly from their earlier paleo-configuration [9]. The resulting complex evolutionary patterns of the river drainages in Yunnan Plateau make it an especially interesting region for exploring the phylogeography of aquatic species.

Chu [12] and Chu and Chen [13] illustrated the relationship of the six river systems in Yunnan Plateau, based on similarity of genera and suggested that the six river systems can be divided into two groups: Jinsha-Nanpan-Red and Mekong-Salween-Irrawaddy (Figure S1). In addition, Several molecular phylogenetic studies demonstrated that the evolutionary history of some fish species (e.g., glyptosternoid fishes, subfamily Schizothoracinae) are associated with the uplifts of the Tibetan plateau and the concomitant river rearrangements (mostly river capture) [14], [15], [16]. These studies also suggest that the Mekong River had a close relationship with the Salween River, while the Red River had closely related with Jinsa River, Nanpan River and Beipan River. However, because these studies were performed on the fish species that mainly inhabit the cold water of the upper river systems, it is difficult to know whether the evolutionary patterns are actually fitted to the basins of the Salween, Mekong and Red Rivers in the southwest of the Yunnan Plateau.

A recent molecular study on the phylogeography of Yunnan spiny frog (*Nanorana yunnanensis*) suggested that the basins of the Salween, Mekong and Red Rivers in the southwest of the Yunnan Plateau have experienced little change since the early Pliocene, excepting in their upper portions near southeastern Tibet [17]. Previous geological studies on Quaternary sediments of these rivers, however, suggest that the mainstreams of the Salween, Mekong and Red Rivers roughly formed or joined up during the Kunlun-Yellow River Movement (KYM) (1.1–0.6 MYA) in the mid-Pleistocene [9], [10], [18]. On the surface, the results of the phylogeography of the Yunnan spiny frog seem inconsistent with the results of the geological studies of this region, suggesting the historical rearrangement of these rivers is still poorly understood.

To date, studies examining the phylogeography at intraspecific level or between closely related fish species inhabiting these drainages that can be used to evaluate the change to the drainage configurations in this region are lacking. In the present study, we examined the maternal phylogeographical pattern of *Poropuntius huangchuchieni* (Tchang, 1967) in the basins of the Mekong and Red Rivers and *P. opisthoptera* (Wu, 1977) in Salween River basin to investigate the association between the changes among the three river drainages and the genetic differentiation of freshwater fish species. *P. huangchuchieni*, a medium sized (about 130–300 mm) economic freshwater cyprinid fish, is widely distributed in the subtropical and tropical waters of Mekong and Red River basins [19], [20]. The individuals of *P. huangchuchieni* dwell where the river flow becomes retarded, subsisting on a diet of plankton and mosses attached to stones or rotted wood.

In this study, we used the sequences of the mtDNA control region to estimate the genetic differentiation and phylogeographical patterns of *P. huangchuchieni*. To examine the taxonomic status of *P. opisthoptera*, we also determined the phylogenetic relationships between *P. huangchuchieni* and *P. opisthoptera*. Accordingly, the main objectives of this study are (1) to examine the population genetic structure and the demographic history of *P. huangchuchieni*; (2) to propose a historical biogeography and climate hypothesis to accommodate the phylogeographic patterns of lineages within *P. huangchuchieni*; and (3) to determine the taxonomic implications for *P. opisthoptera*.

## Results

### Sequence Variation and Genetic Diversity

After alignment, 965 bp DNA sequences of the complete control region were obtained from all *P. huangchuchieni* specimens. The control region sequences yielded 126 variable sites of which 100 were parsimony informative, defining 126 haplotypes (GenBank Access Number: KC567019-KC567146). The overall nucleotide diversity (π) and haplotype diversity (*h*) of all in-group sequences was 0.0286±0.0139 and 0.9856±0.0020, respectively. In total, 1140 bp DNA sequences of the complete *Cyt b* gene were determined from 20 in-group individuals, and these 20 *Cyt b* sequences contained 117 variable sites of which 87 were parsimony informative, further defining 18 haplotypes (GenBank Access Number: KC567001–KC567018).

### Phylogenetic Analysis

**Figure 1:**
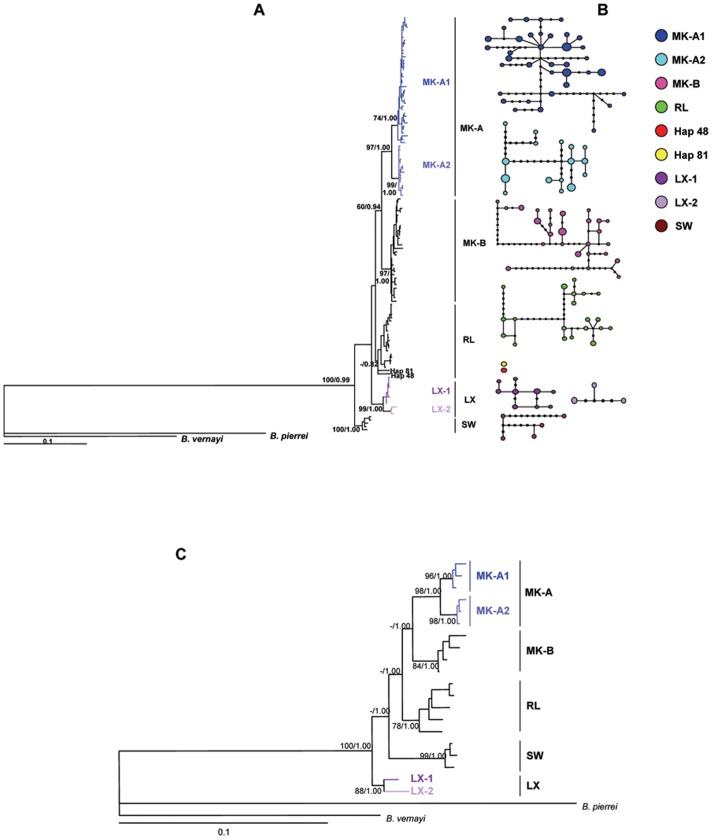
Phylogenetic trees reconstructed based on mitochondrial control region sequences of all haplotypes (A), the combined sequences of 20 haplotypes (C), and the haplotypes network analysis of the 126 haplotypes (B). (A) ML tree reconstructed based on mitochondrial control region sequences of all haplotypes under HKY+I+G model. Numbers on major nodes represents bootstrap values after 1,000 replications. If bootstrap values were less than 50%, they were defaulted. Trees were rooted by *H. pierrei* and one *H. vernayi*. (B) Haplotypes networks conducted based on the 126 haplotypes. Circle size is proportional to haplotype frequency. The number of black dots on line connected haplotypes represents mutation steps between haplotypes; when the mutation step is 1, it was defaulted. (C) 50% majority-role consensus tree inferred from ML and Bayesian analysis of combined sequences of 20 haplotypes under GTR+I+G model. Numbers at nodes represent the posterior probability for Bayesian analysis and bootstrap value for maximum likelihood (ML) analysis. If the bootstrap values were less than 50%, they were defaulted. Trees were rooted by *H. pierrei* and one *H. vernayi*.

ML and Bayesian phylogenetic analysis of the 126 haplotypes identified two major phylogroups, containing five highly independent lineages with strong statistical support (Fig. 1A). One group, named lineage SW, clustered all of the *P. opisthoptera* individuals from the Salween River, which was at the most basal position of the tree. The other group contained all *P. huangchuchieni* individuals, which formed four major evolutionary lineages: MK-A, MK-B, RL and LX (Fig. 1A).

**Figure 2:**
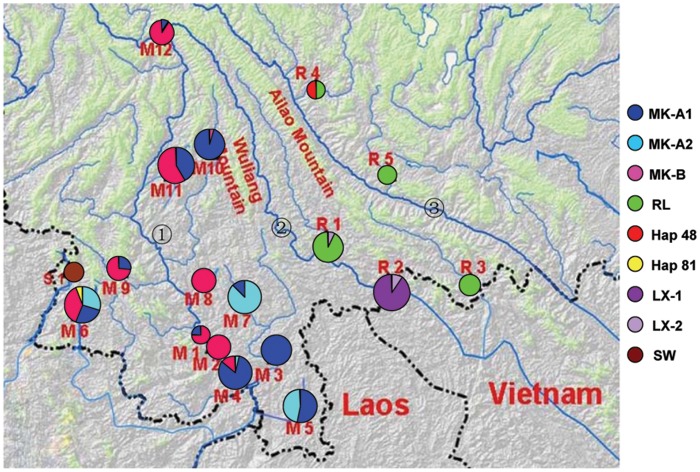
Geographic distribution of sample sites and the frequency of lineages and sublineages at each site. Circle area is proportional to the sample size. 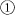
: Mekong River; 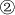: Lixian River; 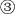: Red River.

Lineages of MK-A and MK-B were represented by nearly all of the haplotypes (except of Hap 81) from the Mekong River System, and co-occurred in most sample sites in the Mekong River System (Table 1). The haplotypes from Luosuo River (M3), Nanla River (M5) and Puwen River (M7) in the Mekong River system were only found in lineage MK-A, and the haplotypes from Menghan (M2) and Dazhong River (M8) were only found in MK-B (Table 1). The RL lineage contained the haplotypes from Amo River (R1), Tengtiao River (R3), Lvzhi River (R4) and Hedi River (R5) in the Red River System. Interestingly, two haplotypes–Hap48 from the Lvzhi River within the Red River System and Hap81 from Nanlei River within the Mekong River System–fell outside the main cluster of lineage RL and located at the basal position within lineage RL (Table 1). Lineage LX was comprised of all of the haplotypes from Lixian River (R2) and two haplotypes from Amo River (R1) (Table 1). Within MK-A, there were two sublineages, which were named as MK-A1 and MK-A2. MK-A1 was distributed in most of the sample sites (10 of 12 sample sites) of Mekong River System, whereas MK-A2 was distributed in only 4 sample sites (Figure 2). Lineage LX likewise contained two sublineages, which we named LX-1 and LX-2. LX-1 was only located in the Lixian River, while LX-2, which contained three haplotypes, was found in both the Lixian and Amo River (Figure 2).

**Table 1:** Sampling information and size in each lineage, as well as haplotype, and nucleotide diversity of populations based on mtDNA D-loop sequences.

Sitecode	Site description	Longitude	Latitude	Number of individuals ineach lineage						*h*	π
				Total	MK-A	MK-B	RL	LX	SW		
M1	Mekong River; Jinghong	100.805 E	22.011 N	4	1	3				**–**	**–**
M2	Mekong River; Menghan	100.925 E	21.845 N	11		11				0.8333±0.1002	0.0062±0.0036
M3	Luosuo River, a tributary of MekongRiver	101.262 E	21.927 N	28	28					0.8492±0.0538	0.0045±0.0026
M4	Mekong river; Guanlei	101.123 E	21.688 N	28	24	4				0.9200±0.0339	0.0088±0.0047
M5	Nanla River, a tributary of Mekong River	101.560 E	21.437 N	29	29					0.8941±0.0382	0.0154±0.0079
M6	Nanlei River, a tributary of MekongRiver	99.608 E	22.369 N	34	19	13	2*			0.8699±0.0442	0.0271±0.0135
M7	Puwen River, a tributary of Luosuo River	101.067 E	22.505 N	24	24					0.7935±0.0566	0.0084±0.0045
M8	Dazhong River, a tributary of MekongRiver	100.574 E	22.557 N	3		3				–	–
M9	Hei River, a tributary of Mekong River	100.251 E	22.636 N	11	3	8				0.7091±0.0827	0.0191±0.0104
M10	Weiyuan River, a tributary of MekongRiver	100.709 E	23.485 N	21	20	1				0.8857±0.0473	0.0076±0.0041
M11	Mengjia River, a tributary of MekongRiver	100.310 E	23.594 N	41	17	24				0.9207±0.0258	0.0220±0.0110
M12	Mekong River, Manwan	100.496 E	24.525 N	10	1	9				0.8889±0.0754	0.0147±0.0081
R1	Amo River, a tributary of Lixian River	101.753 E	23.114 N	27			25	2		0.9316±0.0245	0.0118±0.0062
R2	Lixian River, a tributary of Red River	102.293 E	22.574 N	21				21		0.9053±0.0441	0.0058±0.0032
R3	Tengtiao River, a tributary of LixianRiver	103.155 E	22.604 N	6			6			–	–
R4	Lvzhi River, a tributary of Red River	101.582 E	24.243 N	2			2#			–	–
R5	Hedi River, a tributary of Red River	102.329 E	23.425 N	4			4			–	–
S1	Nanka River, a tributary of SalweenRiver	99.385E	22.574N	5					5	–	–

* Hap 81;

#one of the individuals represented Hap 48.

In the phylogenetic trees based on the combined sequences of control region and the *Cyt b* gene, the five major lineages could be clearly detected (Fig. 1C), corresponding to those defined in the control region trees. Compared with the control region trees, the placement of Lineages SW and LX were slightly different; LX was placed at the most basal position and SW at the second basal position in the combined sequence trees. Based on the combined sequences, the positions of other three lineages were recovered with strong supports.

### Population Genetic Structure

In total, among the 5 lineages, 126 haplotypes formed 9 unconnected haplotype networks at the 95% connection limit (Fig. 1B). MK-A was divided into two unconnected sublineages, MK-A1 and MK-A2. LX was also unconnected and formed two independent networks, LX-1 and LX-2. RL was divided into three unconnected networks, where Hap 48 and Hap 81 formed two independent networks, while all the other haplotypes formed an integrate network.

The geographical structure at the river systems levels was determined using AMOVA analysis. Once all populations were grouped into the three river systems (*F*st = 0.650, *F*ct = 0.331, *P*<0.0001), 33.09% of the variation was between the different river systems and 31.91% of the variation was between populations within the same river systems.

### Divergence Time Estimates

In conducting the relative rate test, the null hypothesis of rate constancy could not be rejected for all lineages pairs. Accordingly, mtDNA divergence could instead be used to calculate the divergence times of the inferred mtDNA lineages. We calculated the net between–lineage mean distances using 20 *Cyt b* sequences, with values ranging from 0.016 to 0.033 among the inferred lineages (Table 2); the net between–lineage mean distances estimated using all the control region sequences were between 0.017–0.030 (Table 2). We also calculated the net between–lineage mean distances based on the same 20 specimens of control region sequences with values from 0.013 to 0.038 (results not shown) among the inferred lineages. Accordingly, it could be concluded that the evolutionary rate of the control region sequences was similar to that of the *Cyt b* gene among the five major lineages of *P. huangchuchieni.*

**Table 2:** Genetic distances among 5 major lineages based on *p* distance. Net genetic distances estimated based on the *Cyt b* data set are above the diagonal, net genetic distances estimated based on the D-loop data set are below the diagonal.

	MK-A	MK-B	RL	LX	SW
MK-A		0.016±0.003	0.019±0.004	0.024±0.004	0.033±0.005
MK-B	0.018±0.003		0.017±0.003	0.023±0.004	0.029±0.004
RL	0.019±0.004	0.016±0.003		0.024±0.004	0.028±0.004
LX	0.025±0.004	0.022±0.004	0.021±0.004		0.024±0.004
SW	0.035±0.005	0.037±0.006	0.026±0.005	0.031±0.006	

The most recent common ancestor (TMRCA) of all the ingroup sequences was dated back to around 1.57 MYA (95% CI = 1.03–2.21). Meanwhile, the estimated divergence time of the lineage LX from lineages MKs and RL was estimated at 1.28 MYA (95% CI = 0.89–1.70); the diversification time of lineage RL from lineages MKs was 1.11 MYA (95% CI = 0.79–1.46). The TMRCA of lineages MKs and MK-A were dated 0.96 MYA (95% CI = 0.67–1.27) and 0.73 MYA (95% CI = 0.49–0.99), respectively.

### Population Historical Demography

**Figure 3:**
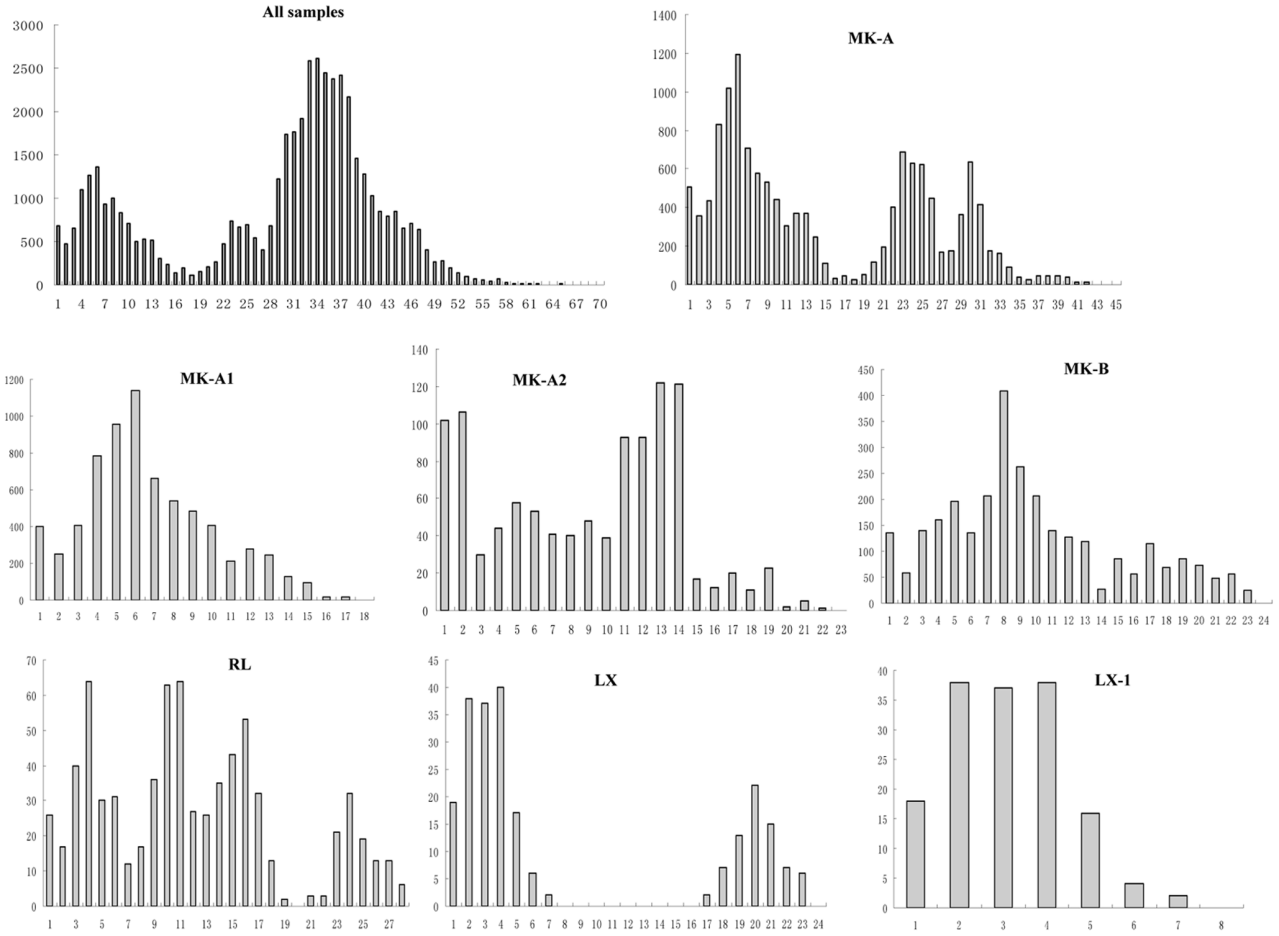
Mismatch distribution analysis of major lineages or sub-lineages of *P. huangchuchieni* based on control region sequences.

The mismatch distribution analysis of Lineages MK-A, RL and LX were multimodal distribution of pairwise differences, and the Fu’s *Fs* values were also not significant, indicating a long term demographic stability in these lineages (Fig. 3). Lineage MK-B displayed a unimodal mismatch distribution and a significant *Fs* value, suggesting a recent population expansion. Lineage SW was excluded from this analysis because of its small sample size. Two sublineages (MK-A1 and LX-1) fitted the expected distributions under the expansion model (Fig. 3). Mismatch distributions for sublineages MK-A1 and LX-1 were unimodal, with *F*s values of −16.48 (*P*<0.01) and −3.48 (*P*<0.05), respectively (Table 3). Neither mismatch distribution nor neutrality tests, however, supported the expectation of population expansion for sublineage MK-A2. Given the small sample size of sublineage LX-2, the detection of population expansion was not performed. With the estimated tau value (τ) and a generation time of one year, the estimated times since population growth for lineage MK-B, and sublineages MK-A1 and LX-1 were respectively dated to around 140 ka, 100 ka, and 60 ka years ago (Table 3).

**Table 3:** Statistics of genetic diversity, tests of neutrality, and demographic parameters estimated for the major lineages and sublineages. These data were defaulted when number of individuals in a population was less than 10.

	No. of individuals	No. of haplotypes	Haplotype diversity	Nucleotide diversity	Tajima’D	Fu’s *F*s	Tau	T(KY)
MK-A	166	54	0.9593±0.0068	0.0110±0.0056	−0.0762	−8.804	–	–
MK-B	77	28	0.9482±0.0097	0.0143±0.0072	0.112	−6.842*	5.449	140
RL	39	26	0.9771±0.0109	0.0183±0.0092	−0.342	−2.828	–	–
LX	22	13	0.9328±0.0299	0.0171±0.0088	−0.040	−0.664	–	–
SW	5	5	–	–	–	–	–	–
MK-A1	119	39	0.9427±0.011	0.0060±0.0032	–1.256	–16.48*	3.906	100
MK-A2	47	16	0.9056±0.020	0.0083±0.0044	–0.536	0.112	–	–
LX-1	17	9	0.8824±0.0513	0.0022±0.0015	–0.912	–3.48*	2.229	60

* *P*<0.05.

### Morphometric Analysis

**Figure 4:**
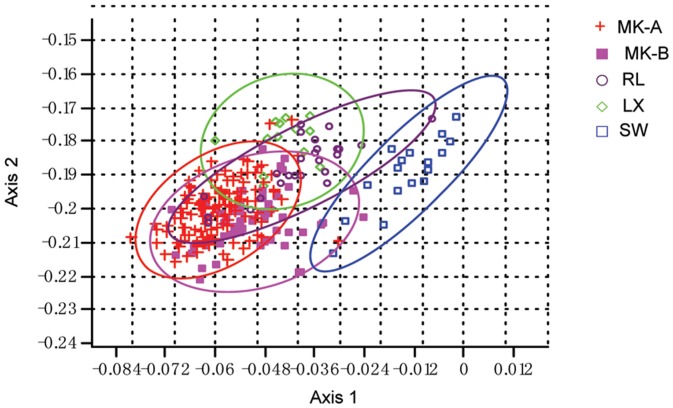
Canonical variate analysis (CVA) based on morphological characters of inferred mitochondrial lineages of *P. huangchuchieni*.

A total of 234 specimens were included in morphological analysis (lineage MK-A: n = 124; lineage MK-B, n = 55; lineage RJ, n = 25; lineage LX, n = 13; lineage SW, n = 18). The Canonical Variates Analysis (CVA) produced a scatter of specimens along the two first canonical axes (Fig. 4). Plotting canonical variables 1 (CV1) and canonical variables 2 (CV2) showed a clear morphometric space among groups. Together, these first two canonical variables collectively explain 83.64% of the total variability between groups (CV1 accounted for 65.09% and CV2 accounted for 18.55%). Meanwhile, 95% frequency ellipses showed an overlap in the scatter of data among the 5 groups, with the exception of lineages MK-A and LX not overlapping with lineage SW (*P. opisthoptera*) (Fig. 4). The CV1 sets lineage SW (*P. opisthoptera*) as having diverged from the other 4 lineages, being then associated with the relative position of the dorsal fin (Table 4). The CV2 sets lineage LX from lineages MK-A and MK-B, and corresponded to the relative heights of body and caudal peduncle (Table 4).

**Table 4:** Factor loadings for the 10 highest measured variables and canonical correlations of the differentiation analysis for the 5 main lineages.

Variable	Axis 1	Variable	Axis 2
X3	**0.5450**	Y4	**0.5534**
X4	**0.5177**	Y7	**0.3923**
X9	–0.3194	Y10	–0.3603
Y1	0.2499	X8	–0.2847
Y7	–0.2415	X1	0.2244
Y10	–0.1909	Y6	0.2212
Y5	–0.1738	X4	0.1940
Y11	0.1628	Y5	0.1864
X2	0.1652	X9	–0.1802
X7	–0.1441	Y1	0.1759

## Discussion

### Taxonomic Implications

The mitochondrial control region phylogenetic tree revealed the basal position of *P. opisthoptera*, which suggested a close relationship with *P. huangchuchieni*. However, the phylogenetic trees based on both the *Cyt b* gene and combined genes all showed the basal position of Lineage LX, and the second basal position of *P. opisthoptera*, indicating that *P. opisthoptera* may instead be a paraphyletic group of *P. huangchuchieni*. While the phylogenetic trees based on different data sets provided somewhat ambiguous results for the taxonomic inference of *P. opisthoptera*, together they firmly supported the notion that *P. opisthoptera* and the four major lineages of *P. huangchuchieni* all originated from a common ancestral stock.

**Figure 5:**
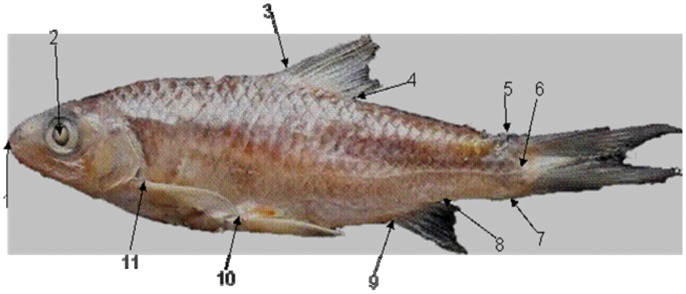
Locations of landmarks on the body surface of *P. huangchuchieni.* Note: 1: Tip of the mouth, junction of premaxilary and ethmoid; 2: Center of eyes; 3: Former basal point of dorsal fin; 4: Posterior basal point of dorsal fin; 5: Upper basal point of tail fin; 6: Centre basal point of tail fin; 7: Abdominal basal point of tail fin; 8: Posterior basal point of anal fin; 9: Former basal point of anal fin; 10: Former basal point of ventral fin; 11: Former basal point of pectoral fin.

The differentiation of *P. opisthoptera* from the four lineages of *P. huangchuchieni* may have resulted from the separation of the Salween River and Mekong River systems (see the next section). At the morphological character levels, the unique evidence that illustrated *P. opisthoptera* as a separate species from *P. huangchuchieni* was the position of the dorsal fin [19], [20]. In *P. opisthoptera*, the distance between the origin of the dorsal fin and the base of tail fin was shorter than that between the origin of the dorsal fin and the posterior end of preopercular bone. In *P. huangchuchieni*, the distance between the origin of the dorsal fin and the base of tail fin was no less than the distance between the origin of the dorsal fin and the posterior margin of eyes (Fig. 5). In our Canonical Variates Analysis (CVA), the position of the dorsal fin greatly contributed to the separation of *P. opisthoptera* from the four major lineages of *P. huangchuchieni*, in which the dorsal fin was placed more anteriorly in the four lineages of *P. huangchuchieni* but more posteriorly in *P. opisthoptera*. However, we detected several individuals in lineages MK-A, MK-B and RL that shared the posterior dorsal fin characteristic with individuals of *P. opisthoptera* (Fig. 4). This finding indicated that the position of dorsal fin was a continuous character and as such could not be used as diagnostic in order to discriminate the two species. Furthermore, this finding suggested that *P. opisthoptera* is not an effective species, but a paraphyletic group of *P. huangchuchieni*-a supposition consistent with our molecular analyses. Accordingly, our molecular and morphometric results both support that *P. opisthoptera* should be considered one of the major lineages of *P. huangchuchieni*.

### Phylogeny and Phylogeographic Pattern in Southwestern Yunnan Plateau

Both our phylogenetic and population structure analyses provided clear evidence for five highly differentiated lineages within *P. huangchuchieni* (Fig. 1). Together, the five major lineages were almost constrained to the three different river systems, in which MK-A and MK-B were in Mekong, RL (except Hap81 which is from the Mekong River System) and LX in the Red River, and SW in the Salween River System. The highly structured phylogeographic pattern of *B. huangchuchieni* is consistent with the current drainage patterns in the Yunnan Plateau. The long-term geographic isolation was clearly an important factor in shaping the current genetic structure and distribution of the major lineages of *P. huangchuchieni* in the three river systems.

Though highly differentiated among the major lineages, the basal topology of our phylogenetic reconstruction is poorly supported. These results might then suggest that the differentiation events among the five major lineages occurred quite rapidly; and the splits of some lineages (i.e. Lineage LX, Lineage RL, Lineage MK-A and Lineage MK-B) likely took place within a comparatively very short period. Our analysis dated the divergence times among the five lineages of *P. huangchuchieni* to 0.96–1.57 MYA. The dated splits times among the major lineages of *P. huangchuchieni* in three river systems therefore can be tentatively associated to the Kunlun-Yellow River Movement (KYM) some 1.10–0.60 MYA [18], which is approximately consistent with the deformation events of the Yunnan Plateau [9] and the continuingly complex development of watersheds and watercourses during the intense Pleistocene geologic movements [9], [10], [18], [21], [22], [23].

The differentiation of LX in the Red River System deserves particular attention, because it is located at the basal position of the molecular phylogenetic trees (Fig. 1C). The Lixian River is an intermediate drainage that had linked the Mekong and Red Rivers in recent bio-geographic history [24]. This pattern was further supported by phylogeographic analysis of another freshwater fish species *Opsarius pulchellus* (our currently unpublished data). However, the phylogenetic analyses based on both the control region and the combined sequences taken together provided consistent evidence supporting LX’s divergence before the separation of the Mekong and Red River systems (Fig. 1A, 1B and 1C). We then conjectured that this discrepancy may be caused by the special geologic or climate changes in the recent evolutionary history, which might fragment the population of lineage LX. However, this explanation is only a hypothesis due to the relatively poor understanding the paleo-geologic and paleo-climatic changes in this region. The driving factors of lineage LX’s differentiation are still unclear. Clearly, further investigation on the geologic research and phylogeography of other aquatic species might help to elucidate this question more fully.

Clearly, the particular evolutionary history of the three river systems revealed by our results is partially consistent with the known phylogeography of the fish species (e.g., glyptosternoid fishes, subfamily Schizothoracinae) inhabiting in the upper portions of these drainages near southeastern Tibet [14], [15], [16], which all suggest a close relationship between Salween River and Mekong River. Additionally, our results are inconsistent with the phylogeography of *Nanorana yunnanensis*, which proposed that Salween, Mekong and Red River basins in the western part of Yunnan Plateau experienced little change since the early Pliocene [17]. However, the deep river valleys were shown to have most likely acted as barriers for the montane mammal *Apodemus ilex* [26]. This discrepancy may potentially be due to the more complex geographic patterns that resulted from the evolution of the drainage systems, all of which continued to play different roles in regards to the genetic structure of different animals.

### Population Differentiation within River Basins in Southwestern Yunnan Plateau

The population differentiated in Mekong and Red River Systems after the KYM vicariant event. For example, the lineage MK-B and MK-A which split into two sublineages, MK-A1 and MK-A2, in Mekong River System; the lineages RL and LX which split into two sublineages, LX-1 and LX-2, in the Red River System (Fig. 1). Independent geological or climatic evidence often serves to suggest that distinctive mtDNA phylogroups may have diverged in allopatry [25]. In addition, most patterns in mtDNA surveys also reveal the secondary admixture between allopatrically evolved phylogroups [25].

The estimated split time between the two lineages in Mekong River System was dated back to about 0.96 MYA. Lineages MK-A and LX diverged into two highly differentiated sublineages: MK-A1 and MK-A2 within the lineage MK-A, and LX-1 and LX-2 within the lineage LX (Fig. 1A, 1B and 1C), implying a long term genetic isolation. Additionally, the unimodal mismatch distributions and the neutrality tests (Fu’s *Fs*) both suggest the population expansion event in lineage MK-B, sublineages MK-A1 and LX-1, estimated approximately 0.06–0.14 MYA and thus consistent with the largest glacial retreat that occurred 0.175 MYA [30], [31], [32]. Additionally, MK-A and MK-B have an overlapped distribution in most localities (Fig. 2; Table 1). Also, a few sublineages were detected co-occurring in sympatry within both the Mekong and Red River systems: i.e., haplotypes of sublineage MK-A1 and MK-A2 coexisted in some sample sites (e.g. M5, Mengla River; M7, Puwen River) in the Mekong River System; lineages LX and RL co-occurred at the Amo River within Red River System (Fig. 2). This pattern suggests secondary contact occurred after the initial divergence in the divergent lineages and sublineages within each drainage system. These demographic patterns might suggest that the sudden global cooling could then potentially explain a restricted gene flow and make the ancestral populations of lineages or sublineages restricted into different refugia, which followed a population expansion event and secondary admixture after the long term isolation between the differentiated lineages/sublineages in each drainage system. The differentiation, recent population expansions and secondary contact of lineages/sublineages within the Mekong and Red River Systems was also found in the phylogeographic analysis of another freshwater fish species *Opsarius pulchellus* (our currently unpublished data). These comparative findings suggest that the long term isolation between lineages caused by climate factors is likely the underlying mechanism of the observed population differentiation between the lineages of *P. huangchuchieni* in the Mekong and Red River Systems. The pattern of population expansion and secondary contact might well be attributable to the climatic oscillations during the extensive glacial period in the Pleistocene and to postglacial population expansions. The diverse environments in the southwestern Yunnan Plateau may have created refugia during glaciations which accordingly acted as barriers to subsequent expansion. However, in some lineages/sublineages (i.e. lineage RL, sublineage MK-A2), we detected a population equilibrium. This pattern could also be attributed to the diverse environments in the southwestern Yunnan Plateau and climatic oscillations during glacial periods, allowing some lineages to expand while others remained stable [33]. In addition, phylogeographic studies on *Opsarius pulchellus* (our currently unpublished data), some frogs which must live near rivers, like the montane frog [33], Yunnan spiny frog [17], and even the montane mammal *Apodemus ilex* [26], have similar population expansion and secondary contact patterns within the Mekong and Red River basins as well as within the Hengduan Mountains. On the whole, these evidences further suggest that the Pleistocene climatic changes affected the distribution and genetic structure of animals inhabiting the region.

During our analysis, we also recognized a multimodal mismatch distribution within all samples of *P. huangchuchieni* in this study (Fig. 3). This could potentially be attributed to a pattern of long term demographic stability and a large effective population size in the recent evolutionary history. These results reveal that population of *P. huangchuchieni* might have been widely distributed in the drainage and keep a high gene flow in a large population for a long period of time, even when the three river systems still interconnect as a single drainage. This pattern was further supported by the demographic stability and equivalent genetic diversity of three major lineages (lineages MK-A, RL and LX) in Mekong and Red River Systems (Table 3, Fig. 3). Because a small population colonized a new region, a recent population expansion event, characterized by a lower genetic diversity and a Poisson-like mismatch distribution, would be likely be found in the colonized region [27]. In this study, we found no evidence of a recent expansion event in the major lineages (except lineage MK-B) of *P. huangchuchieni* in these drainages. As such, it can be inferred that the population of *P. huangchuchieni* dispersed in the three river systems with a large population sizes and differentiated rapidly (Figs. 1, 3).

The large population sizes of these lineages in Mekong and Red River Systems might play a critical role in maintaining the higher levels of diversity and the evolution of lineages within *P. huangchuchieni* (Table 3), such as the prevention of the complete lineage sorting among drainages [25]. Therefore, we found two highly differentiated haplotypes (Hap 81 and Hap 48) in this study (Fig. 1), which were from peripheral populations of Mekong (M6) and Red (R4) River Systems, fall outside the main cluster of lineage RL and located at the basal position within lineage RL (Fig. 1A). The haplotype in the Mekong River System clustered into the lineage RL may have resulted from incomplete lineage sorting, an event that is more likely to occur within large effective populations which can accommodate more polymorphisms than a small sized population [28]. This situation likewise mainly occurs when population divergence is recent [29]. Accordingly, the large effective population of *P. huangchuchieni* and the recent Pleistocene differentiation of lineages are responsible to the incomplete lineage sorting between the Mekong and the Red River Systems.

## Conclusion

The results of our study showed that the populations of *P. huangchuchieni* in three river systems (the Salween, Mekong and Red River Systems) were highly differentiated into five main lineages. Likewise, our results revealed that *P. opisthoptera* (lineage SW) was not a separate species but a paraphyletic group of *P. huangchuchieni*. The accumulated evidence suggests that the differentiation of the major lineages within the three river systems may be associated with the drainages’ division of the intense uplifting of Qinghai-Tibetan Plateau during the KYM event. The genetic differentiation within river basins and recent demographical expansions occurred in some lineages and sublineages are consistent with the Pleistocene climatic oscillations. Additionally, our results also suggest that the population of *P. huangchuchieni* may have been widely distributed in the southwest region of Yunnan Plateau, maintaining a high gene flow in a large, stable population in the recent evolutionary history, which might be responsible to the incomplete lineage sorting. Additional studies on other aquatic species would likely help to clarify some of our preliminary findings and greatly add to our understanding on how geographic events and paleo-climate changes shape the genetic structure of aquatic populations in areas like the Yunnan Plateau.

## Materials and Methods

### Ethics Statement

*P. huangchuchieni* is a common economic fish. All specimens and muscle tissues were bought from local fish dealers. The specimens used in this study were dead. Samples were obtained following the regulations for the implementation of China on the Protection of Wild Animals (Presidential Decree [2004] No. 9).

### Sample Collection

A total of 304 specimens of *P. huangchuchieni* and 5 individuals of *P. opisthoptera* were obtained from 18 geographic localities, covering most of the distribution area of *P. huangchuchieni* in China (Table 1; Fig. 2). As outgroups, we included one individual of each *Hypsibarbus pierrei* and *Hypsibarbus vernayi*, collected from a tributary of the Mekong River. Their species identities were confirmed using the morphological features outlined in previous reports by [19]. All specimens were preserved in 95% ethanol. Voucher specimens were deposited in the Museum of Yunnan University.

### DNA Extraction, PCR Amplification, and Sequencing

Total genomic DNA was extracted from muscle tissues using the standard phenol-chloroform extraction method. The complete sequence of the mitochondrial control region was amplified with the primers used by Gilles *et al.* [34]. Afterward, the complete sequence of the mitochondrial cytochrome *b* gene (*Cyt b*) was amplified for a subset of samples, selected based on the topology of the control region sequences, using the primers outlines by Xiao *et al.* [35]. PCR amplifications were carried out in 50 ul reaction mixture containing 5 ul 10×PCR buffer (TaKaRa, Dalian), 0.2 mM dNTPs, 0.2 uM each primer, with 1.5 U Taq DNA polymerase (TaKaRa) and approximately 50 ng genomic DNA. Reaction condition were 3 min at 95°C, followed by 35 cycles of 1 min at 94°C, 1 min at 57°C (for the control region) or 52°C (for *Cyt b*), and 1 min at 72°C, and 7 min at 72°C. PCR products were purified using the Gel Extraction Mini Kit (Waston BioTechnologies, Shanghai).

PCR products were sequenced in an ABI Prism 3730 (Applied Biosystems) automatic sequencer. The complete sequences of control region were sequenced directly with PCR primers; the 19 complete sequences of cytochrome b were sequenced using PCR primers and two internal primers (L15286 and H15374) employed by Xiao *et al.* [36]. The sequencing reaction conditions were 25 cycles of 96°C for 30 s, 50°C 15 s and 60°C 4 min.

### Statistical Analysis

All nucleotide sequences of the D-loop region, *Cyt b* and combined sequences (both *Cyt b* and D-loop) were aligned using MegAlign in DNAStar 6 (DNASTAR, Madison, USA), and further alignment was confirmed visually in BioEdit 7.0.9 [37]. Protein-coding nucleotide sequences were translated to amino acids to confirm alignment. The sequence polymorphic analysis was performed in MEGA 4 [38].

The control region phylogenetic relationships were reconstructed using Maximum Likelihood (ML) analysis and Bayesian phylogeny reconstruction to identify the evolutionary lineages of haplotypes. The hierarchical likelihood ratio tests criterion (hlRTs) implemented in Modeltest 3.7 [39] was used to determine the evolutionary model that best fit the empirical data. The HKY+I+G [40] model of sequence evolution was used for reconstructing the phylogeny. ML analysis for the control region haplotypes was evaluated using PhyML [41]. Support for nodes among branches was evaluated using non-parametric bootstrapping [42] with 1,000 replicates. Bayesian inference (BI) for both data sets was performed with MrBayes 3.1.2 [43] with four MCMC chains for 10,000,000 generations, saving trees every 100 generations. The first 20% of the generations were discarded as burn-in and posterior probabilities were determined by constructing a 50% majority rule consensus for the remaining trees.

In order to examine whether the combined sequences could be combined into a larger data matrix, we performed a partition-homogeneity (PH) test in Paup 4.0b10 with 1,000 replicates [44]. The PH test suggested that phylogenetic congruence between the *Cyt b* gene and D-loop region could not be rejected (P = 0.34). The phylogenetic relationships based on haplotypes of combined sequences were reconstructed using Maximum Likelihood (ML) analysis and Bayesian inference (BI). The best fitting model according to Modeltest 3.7 was GTR+I+G [40] for the combined sequences. ML reconstruction was performed in Paup 4.0b10 [44], involving a heuristic search, 10 random additional sequences and TBR branch swapping. Estimates of each lineage’ robustness was obtained using 1,000 non-parametric bootstraps. Bayesian inference (BI) was performed with MrBayes 3.1.2 [43] with four MCMC chains for 10,000,000 generations, saving trees every 100 generations. The first 20% of the generations were discarded as burn-in and posterior probabilities were determined by constructing a 50% majority rule consensus for the remaining trees.

A haplotype network was constructed using TCS 1.2.1 [45] to estimate gene genealogies for the control region. The connection limit was fixed at 95% and gaps were treated as a 5th state. When ambiguities (closed loops or ‘stranded’ clades) occurred in the networks, they were resolved using published rules and predictions based on coalescence theory [46], [47].

Genetic structure analysis was performed based on the control region using Arlequin 3.1 [48]. The haplotype diversity (H) and nucleotide diversity (*π*) [49] for each sampled population with a sample size ≥10 and each lineage/sublineage were estimated. A hierarchical analysis of molecular variance (AMOVA) was implemented to assess the significant population structure on different levels. The fixation index *F*st, *Fct* and *Fsc* [50] was used to estimate genetic differentiation at the three river systems levels. Both AMOVA and *F*st analysis were performed using pairwise difference with gamma correction; the significance was assessed by 10,000 permutations with Arlequin 3.1.1.1 [48].

A molecular clock relative rate test [51] was performed in RRTree [52] using all control region sequences. The relative rate test result was not significant (*P*>0.05), indicating the control region sequences fit the molecular clock hypothesis. To relate the genetic differentiation of the major lineages that were defined by genetic structure analysis to tectonic events of Tibet Plateau and adjacent regions, we estimated the divergence times between the major lineages using published nucleotide substitution rates, as there are no specific rates or fossil records available. To estimate the nucleotide substitution rate of the control region sequences in *P. huangchuchieni*, using Mega 4 we calculated the net between-lineage mean distances based on all of the control region sequences, 19 *Cyt b* sequences and 19 control region sequences [38]. The calculated net between-lineage mean distances were very similar between *Cyt b* sequences and the control region sequences (see Results). Generally, a rate of 2% per million years has been calibrated for *Cyt b* genes in multiple bony fishes [53], [54]. Subsequently, the evolutionary rate of 2% per million years, which may potentially be suitable for the control region sequences of *P. huangchuchieni*, was applied to estimate the divergence time among lineages. The times to the most recent common ancestor (TMRCA) of the major lineages and the whole population were estimated using relax-clock molecular dating estimation implemented in the BEAST 1.5.2 [55]. Analyses using the HKY model of nucleotide substitution with gamma distributed rate variation among sites were performed. The Yule speciation method was assumed and the nucleotide substitution rate of 2% was used. Chains were run for 50 million generations, with the first 20% discarded as burn-in. The results were summarized through TRACER 1.5 [56].

The historical demographic expansion events of each evolutionary lineages or sublineages were examined using mismatch distribution, as implemented in Arlequin 3.1 [48]. We compared the observed frequency distribution of pairwise nucleotide differences among individuals. A multimodal distribution of pairwise differences was displayed in populations at a demographic equilibrium or in decline, whereas a unimodal distribution was in populations that had experienced a sudden demographic expansion. Additionally, values of Tajima’ D [57] and Fu’s *F*s [58] were also calculated to seek evidence of demographic expansions in lineages and sublineages. The statistical significance was tested with 5,000 permutations. The population expansion times of some lineages or sublineages were estimated using the equation τ = 2 ut [59], where τ is the mismatch distribution age expansion parameter, u is the mutation rate per generation for the whole sequences, and t is the expansion time in number of generations. The value u was calculated using the formula u = 2 µk, where μ is the mutation rate per nucleotide and k is the number of nucleotides assayed. We assumed a mutation rate per nucleotide of 2% for control region sequences of in-group individuals.

### Geometric Morphometric Analysis

All specimens were placed on their right side, with their body posture and fins teased into a natural position. Images of 254 fish specimens (including 237 specimens of *P. huangchuchieni* and 17 specimens of *P. opisthoptera*) were collected using a digital camera at 11 landmarks (see Fig. 5) were digitized to describe the body shape of the fish using TpsDig2 [60]. Morphometric and statistical analyses were conducted in PAlaeontological STatistics (PAST) 1.81 [61]. The first step of each analysis was a two-dimensional procrustes to separate shape from size and eliminate variation in the position and orientation of the specimens. For comparison with results obtained from the mitochondrial marker, all groups were defined according to the lineages of mtDNA. The standardized data underwent canonical variate analysis (CVA) to estimate the discrimination among lineages. Population centroids with 95% confidence ellipses derived from the CVA were used to visualize the relationships among individuals of the lineages.

## Supporting Information

Figure S1 **The six main rivers distribute in Yunnan Plateau, China.** The six rivers are divided into two groups, Jinsha-Nanpan-Red and Mekong-Salween-Irrawaddy, by the red lines.
(DOC)
