# Supplementary material for: Molecular Phylogeography and Evolutionary History of Poropuntius huangchuchieni (Cyprinidae) in Southwest China
Source: PLoS One. 2013 Nov 25;8(11):e79975. doi: 10.1371/journal.pone.0079975 (PMC3839932; doi:10.1371/journal.pone.0079975)

Figure S1: The six main rivers distribute in Yunnan Plateau, China. The six rivers are divided into two groups, Jinsha-Nanpan-Red and Mekong-Salween-Irrawaddy, by the red lines.


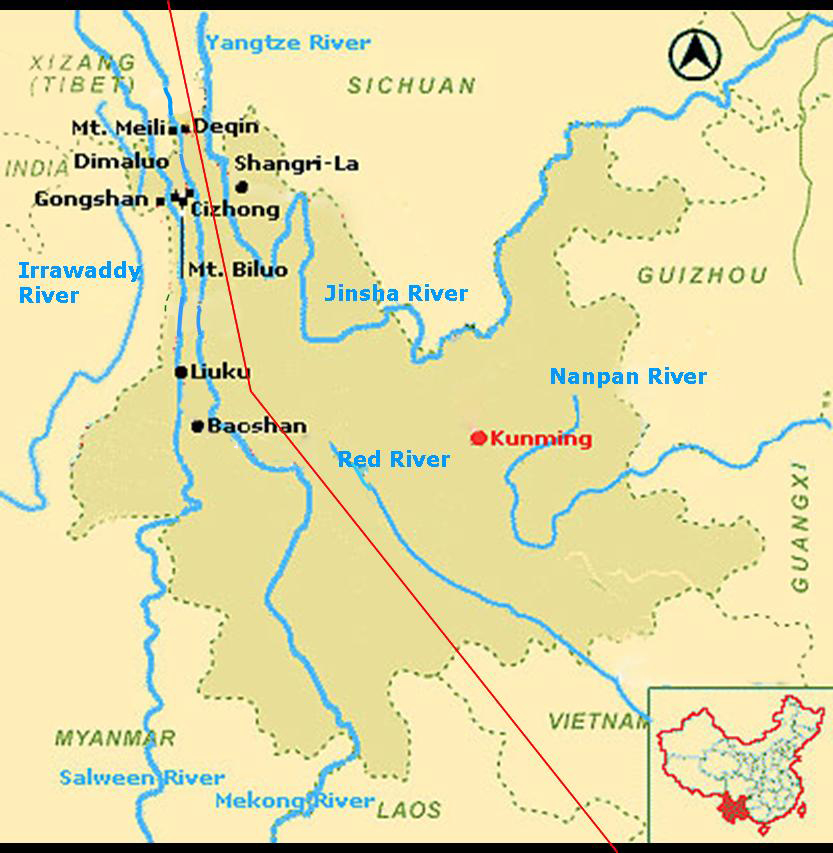

Supplement: Figure S1 — The six main rivers distribute in Yunnan Plateau, China. The six rivers are divided into two groups, Jinsha-Nanpan-Red and Mekong-Salween-Irrawaddy, by the red lines. (DOC) [file pone.0079975.s001.doc]
